# Pharmacological Inhibition of O-GlcNAcase Does Not Increase Sensitivity of Glucocorticoid Receptor-Mediated Transrepression

**DOI:** 10.1371/journal.pone.0145151

**Published:** 2015-12-15

**Authors:** Peter J. Stivers, Lauren Harmonay, Alexandra Hicks, Huseyin Mehmet, Melody Morris, Gain M. Robinson, Peter R. Strack, Mary J. Savage, Dennis M. Zaller, Izabela Zwierzynski, Philip E. Brandish

**Affiliations:** 1 Merck & Co., Inc., Boston, Massachusetts, United States of America; 2 Merck & Co., Inc., Rahway, New Jersey, United States of America; University of Ulm, GERMANY

## Abstract

Glucocorticoid signaling regulates target genes by multiple mechanisms, including the repression of transcriptional activities of nuclear factor κ-light-chain-enhancer of activated B cells (NF-κB) though direct protein-protein interactions and subsequent O-GlcNAcylation of RNA polymerase II (pol II). Recent studies have shown that overexpression of *O*-linked β-*N*-acetylglucosamine transferase (OGT), which adds an *O*-linked β-*N*-acetylglucosamine (O-GlcNAc) group to the C-terminal domain of RNA pol II, increases the transrepression effects of glucocorticoids (GC). As *O*-GlcNAcase (OGA) is an enzyme that removes O-GlcNAc from O-GlcNAcylated proteins, we hypothesized that the potentiation of GC effects following OGT overexpression could be similarly observed via the direct inhibition of OGA, inhibiting O-GlcNAc removal from pol II. Here we show that despite pharmacological evidence of target engagement by a selective small molecule inhibitor of OGA, there is no evidence for a sensitizing effect on glucocorticoid-mediated effects on TNF-α promoter activity, or gene expression generally, in human cells. Furthermore, inhibition of OGA did not potentiate glucocorticoid–induced apoptosis in several cancer cell lines. Thus, despite evidence for O-GlcNAc modification of RNA pol II in GR-mediated transrepression, our data indicate that pharmacological inhibition of OGA does not potentiate or enhance glucocorticoid-mediated transrepression.

## Introduction

Glucocorticoids are effective in inflammatory diseases and blood cancers. [1, 2]. However, steroid insensitivity among asthmatics represents a large unmet medical need [3–5]. Glucocorticoids act by binding to the glucocorticoid receptor (GR), which in turn mediates anti-inflammatory responses in part by binding to nuclear factor κ-light-chain-enhancer of activated B cells (NF-κB) to inhibit transcriptional activity, known as “transrepression” [1]. The large subunit of RNA polymerase II (pol II) contains a unique conserved YSPTSPS heptad repeat in the C-terminus, and transcriptional activation involves the phosphorylation of the heptad at serine-2. Studies by Yamamoto’s group [6, 7] demonstrate that inhibition of NF-κB by GR is caused by its interference of phosphorylation at serine-2 on pol II C-terminal domain (CTD). It has also been suggested that phosphorylation of serine at position 5 of the CTD plays a role in regulation of pol II activity [8–10]. O-GlcNAcylation of serine-5 of the CTD and phosphorylation of serine-5 by a specific CTD kinase from the general transcription factor TFIIH are likely mutually exclusive events and thus a reciprocity exists between phosphorylation and O-GlcNAcylation [8, 11]. Li et al.’s data suggest that the ligand bound GR recruits *O*-linked β-*N*-acetylglucosamine transferase (OGT), which positions an O-GlcNAc on threonine-4, blocking phosphorylation of the pol II transcriptional activation sites [12]. In addition to the effects of OGT, these reversible modifications of serine and threonine residues are also regulated by *O*-linked β-*N*-acetylglucosaminease (OGA) [9, 13, 14]. Using luciferase reporter assays, Li’s group demonstrated that OGT overexpression potentiated glucocorticoid dependent, GR-mediated transrepression of NF-κB. Studies by Ranuncolo et al. confirmed that pol II is O-GlcNAcylated by OGT [11]. They found that inhibition of OGT or OGA blocked transcription during preinitiation complex assembly. It was concluded that O-GlcNAcylation is required to form the preinitiation complex on the DNA promoter, but that removal of O-GlcNAc by OGA is required to allow phosphorylation and initiation of transcription. This would suggest that blocking either enzyme would impair transcription. We hypothesized, by association, that the increase in glucocorticoid efficiency following OGT overexpression, as seen by Li’s group, should be similarly observed by the direct inhibition of OGA. This would result in the inhibition of O-GlcNAc removal from pol II, thus impairing transcription. Here, we use thiamet-G, a small molecule inhibitor of OGA that has shown to be highly selective, to block OGA and determine whether the potency or efficacy of prednisolone was altered in any of several in vitro test systems [15–17]. Despite evidence of cellular target engagement of OGA by thiamet-G, there was no evidence of a potentiating effect on GR-mediated transrepression of NF-κB-controlled genes or pro-apoptotic effects in glucocorticoid resistant cell lines treated with dexamethasone (dex). These studies suggest pharmacological inhibition of OGA does not increase sensitivity to glucocorticoid-mediated transrepression.

## Materials and Methods

### Cell Culture

U-937 cells (ATCC) were stably transfected with TNF-α promoter driving β-lactamase (HTS-43) were maintained in RPMI Medium (LifeTech, Carlsbad, CA, USA) containing 10% HI FBS (LifeTech), 25 mM HEPES (LifeTech), 2 mM L-Glutamine (LifeTech), 1mM Sodium Pyruvate (LifeTech), 55 nM β-mercapthoethanol (LifeTech), and 0.8 mg/mL G418 (LifeTech). Cells were cultured at 37°C and 5% CO_2_. When using the cells in the TNF-α trans-repression assay, the assay media contained 5% charcoal-stripped FBS (LifeTech), instead of 10% HI FBS.

Peripheral blood mononuclear cells (PBMCs) were isolated from heparinized human whole blood using a FICOLL-Paque Plus (GE Healthcare, Little Chalfont, United Kingdom) gradient. Cells were then washed and subsequently cultured in RPMI with 10% HI FBS at 37°C and 5% CO_2_. Written informed consent was obtained from the donors under a study protocol reviewed and approved by Western Institutional Review Board.

A549 cells (ATCC) were maintained in DMEM (LifeTech) containing 10% HI FBS. CEM-c1cells (Sigma, St. Louis, MO, USA) were cultured in RPMI-1640 (LifeTech) with 10% HI FBS. CCRF-CEM cells (ATCC) were cultured in RPMI-1640 media with 10% HI FBS. All cells were cultured and maintained at 37°C and 5% CO_2_. The following primary antibodies were used for staining for immunofluorescence: Invitrogen conjugated alexa-fluor 647 RL2 antibody (0.8 μg/mL), mouse IgG1κ isotype control alexa fluor 647, mouse anti-human mAb CD14-V450 (BD Biosciences, San Jose, CA, USA), mouse anti-human CD3-FITC (BD Biosciences)

### Target engagement assays

Target engagement of OGA by inhibitor thiamet-G was assessed by measurement of accumulation of O-GlcNAc in a HTS-43 cell line, monocytes, and T cells. These cells were incubated with the OGA inhibitor for 24 hr at 37°C prior to permeabilization and staining. The cells were stained with Reagent A, containing formaldehyde, for 5 min in the dark at room temperature and then treated for 10 min in the dark at room temperature with BD FACS lysing solution (BD Biosciences). For 30 min in the dark at room temperature, cells were permeabilized with 50 μL Reagent B, containing sodium azide, plus 10 μL Alexa-fluor 647 RL2, for a final concentration of 4 μg/mL. This assay was performed using manufacturing protocols provided in an Intrasure Kit (BD Biosciences). Target engagement assays were run on a BD FACS Canto II flow cytometer (BD Biosciences) and analyzed using GraphPad Prism 5.

For the TNF-α transrepression assay, HTS-43 cells were plated in assay media and exposed to varying concentrations of prednisolone (Sigma), or DMSO control for 30 min. Cells were then stimulated with phorbol 12-myristate 13-acetate (TPA) (Sigma) and lipopolysaccharide (LPS) from Escherichia coli 0127:B8 (Sigma) at concentrations of 5 ng/mL and 0.1 μg/mL, respectively, for 20–24 hr. β-lactamase assay was performed per instruction from a kit provided by Invitrogen. Output signal was measured using an EnVision (PerkinElmer, Waltham, MA, USA) plate reader. Excitation of samples was at 405 nM and detection of blue coumarin and green fluorescein was at 460 nm and 535 nm, respectively. TNF-α promoter activation values were set as a ratio of blue/green fluorescence signal following the cleavage of substrate CCF4-AM by β-lactamase.

### TNF-α production and gene expression profiling in human PBMCs

The purpose of the pilot study was to validate the protocols and establish a potency of prednisolone on a 36-gene panel in LPS-challenged human PBMCs. The gene panel includes genes activated by LPS but transrepressed by prednisolone (IFIHI, IFIT2, IFIT3, IL1A, IL1RN, NFKBIZ, TRAF1, IL6, SOCS3, CXCL2, CD274, CD40, IFN-γ, PTGS2, TNF-α),genes transactivated by prednisolone (FKBP5, GRASP, PER1, TSC22D3, ZBT B16), genes with no robust response (ADORA3, ECHDC3, IRS2, LPL, MGEA5, NR3C1, PFKFB2, TPST1) and housekeeping genes (GAPDH, TUBB, GUSB, HPRT1, PGK1). The main study used the same system validated in the pilot study but measured the potency of prednisolone in the presence of varying concentrations of OGA inhibitor. For the pilot study, human PBMCs were incubated with varying concentrations of prednisolone (10 μM, 100 nM, 10 nM or DMSO control) with LPS concentrations of 0.01 μg/mL and 1 μg/mL, or vehicle. In the main study, human PBMCs were incubated with half log dilutions of prednisolone (starting at 3 μM) and stimulated with 1 μg/mL of LPS in the presence of thiamet-G (1 nM, 10 nM, 100 nM, 1000 nM,) or vehicle.

In both studies, PBMCs were incubated with prednisolone (+/- 10 uM thiamet-G treatment was in main study only) for 30 min at 37°C. Cells were then stimulated with LPS for 2 hr (for gene profiling) or 4 hr (for TNF-α measurement) at 37°C. TNF-α secreted into the culture medium were detected using mesoscale plates (Meso Scale Discovery, Rockville, MD, USA) according to manufacturer protocols and read on SI 6000 plate reader instrument. TNF-α levels were determined using standards provided by the manufacturer. For gene profiling, cells were lysed on ice using buffer RPE provided by Qiagen (Venlo, Netherlands). Cell lysate was sent to Covance Genetics Lab (Seattle, WA) for analysis. Gene expression was analyzed in cell lysates with the NanoString nCounter system (www.nanostring.com) according to the manufacturer’s instructions. Cell lysate was added to 10 μL Reporter CodeSets and 10 μL hybridization buffer. 5 μL Capture ProbeSet was then added and the hybridization mixture incubated for at least 12 hr. The Nanostring nCounter system then purified hybridized mRNA and counted the number of transcripts in each sample. CodeSets were designed by NanoString based on published sequences for the genes analyzed. Raw count data was normalized for each well in three sequential steps: (1) Background correction (2) positive control correction and (3) housekeeping gene correction. The housekeeping genes were GAPDH, TUBB, GUSB, HPRT1, and PGK1.

### Cell line proliferation and viability assays

A549, CCRF-CEM and CEM-c1 T-lymphoblast cells were incubated overnight and treated in triplicate with dexamethasone, dexamethasone plus 50 nM of ridaforolimus (an mTOR inhibitor used as a positive control), or dexamethasone plus 1 μM thiamet-G for 18 hr. Following incubation, Cell Death Detection ELISA kit (Roche, Basel, Switzerland) was used to determine induced cell death. Cells were lysed using an incubation buffer provided by the kit for 30 min at room temperature. Samples were then incubated with incubated buffer (1:10) for 90 min at room temperature. Plates were washed and substrate solution was added and shaken for 20 min at room temperature. The absorbance was measured at 405 nM on the Spectramax. Cell Titer-Glo Luminescent Cell Viability Assay (Promega, Fitchburg, WI, USA) was used for ATP based detection. For cell viability measurements, A549, CCRF-CEM, CEM-c1 T-lymphoblast cells cells were incubated at 37°C overnight and treated with either dexamethasone, dexamethasone plus 50 nM of ridaforolimus, or dexamethasone plus 1 μM thiamet-G for 72 hr. Cell titer glo was added per the Promega protocol for 10 min and samples were read using Victor V3 microplate luminometer after 72 hr of incubation. Cell viability was calculated as a ratio of treated cells to cells not treated with any compound.

## Results

### TNF-α transrepression in HTS-43 Cells

**Fig 1:**
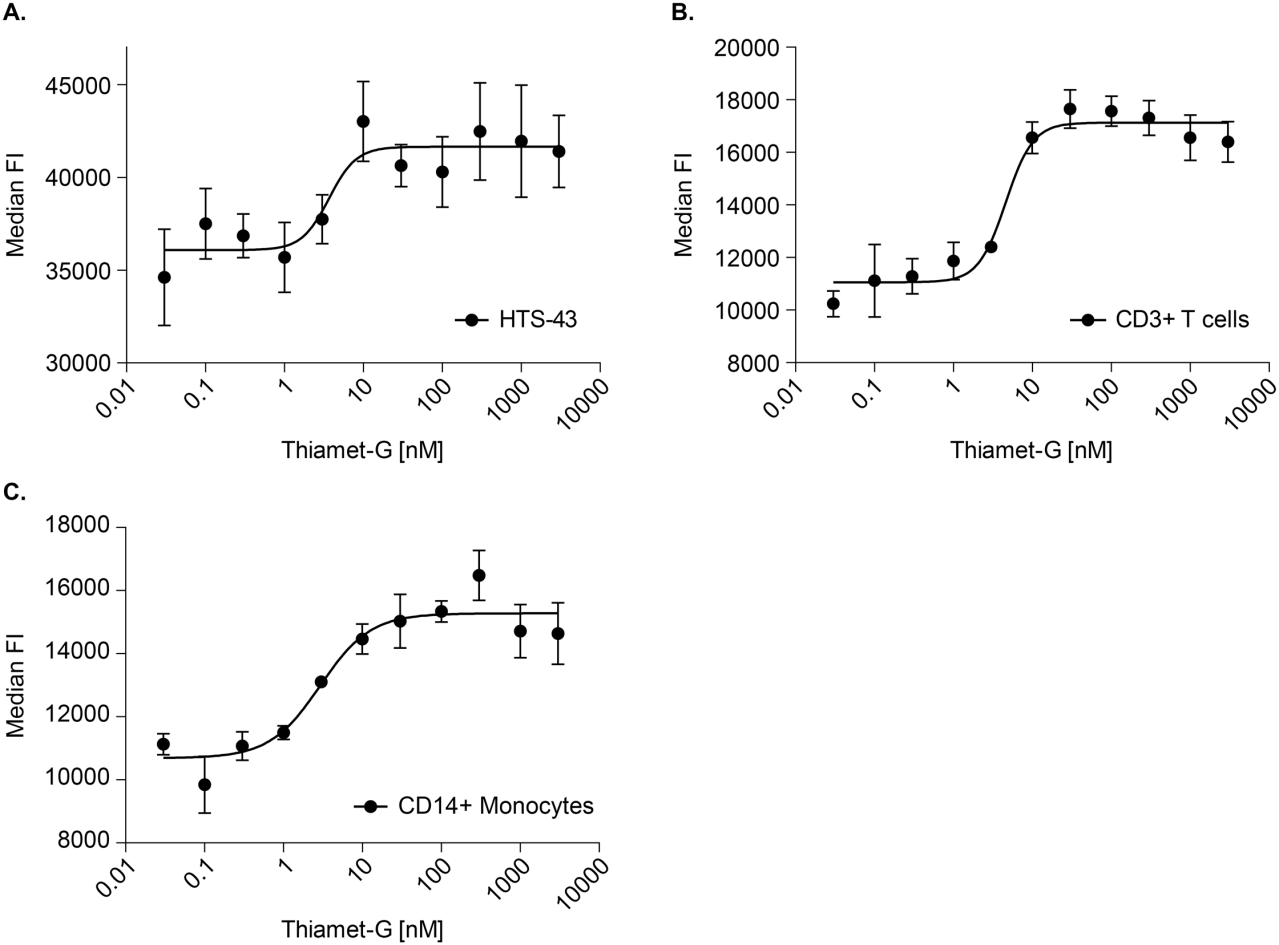
Inhibition of O-GlcNAcase by thiamet -G increases O-GlcNAcylated protein levels in CD3+ T-cells, CD14+ monocytes and HTS-43 cells. (a). HTS-32 cells (CD14+) were incubated overnight at varying concentrations of thiamet-G. The cells were then stained with a custom Alexa-fluor 647 RL2 antibody to detect O-GlcNAac levels via flow cytometry, with units being representative as median fluorescent intensity. (b,c) PBMCs were incubated with varying concentrations of thiamet-G overnight. The cells were stained for monocytes (CD14+/CD3-) and T-lymphocytes (CD3+/CD14-) and then stained with the RL2 antibody for O-GlcNAc detection. Units were measured as mean fluorescence intensity. Data points represent n = 3. Error bars represent standard deviation.

**Fig 2:**
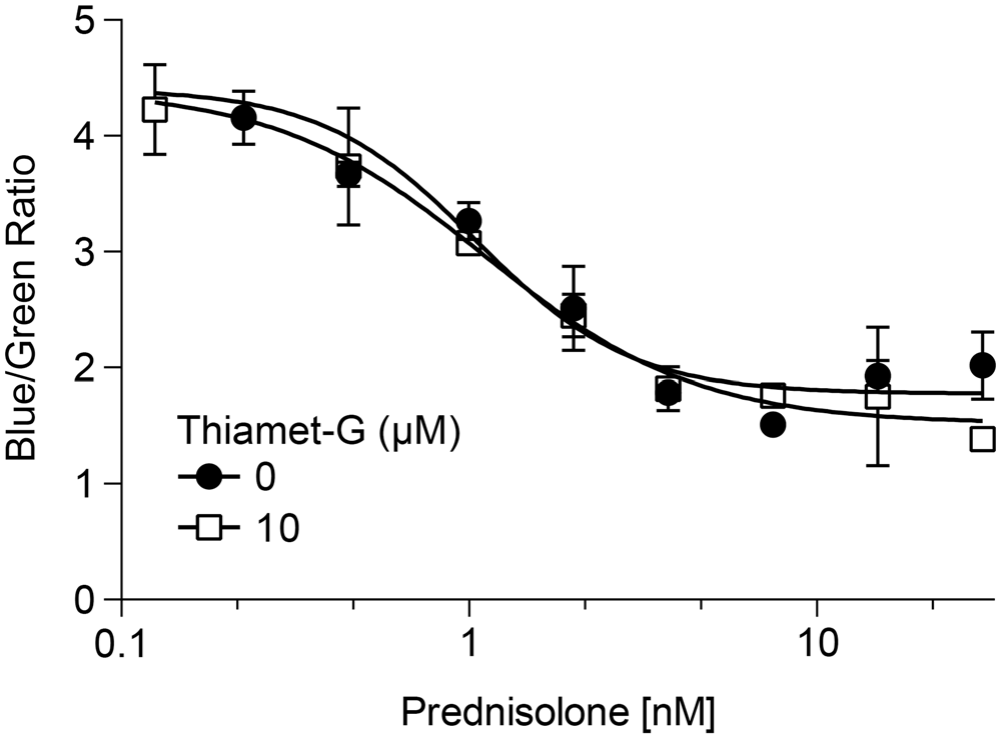
Thiamet-G does not change the potency of prednisolone in HTS-43 cells expressing a TNF-α promoter. HTS-43 reporter cells were cultured with increasing concentrations of prednisolone and/or 10 μM thiamet-G for 30 min at 37°C. The cells were then stimulated with both TPA and LPS at concentrations of 5 ng/mL and 0.1 μg/mL respectively, for 20–24 hr. Activity was measured in a beta-lactamase assay as a blue/green ratio. High ratio value indicates increased TNF-α activity. Data points represent n = 2. Error bars represent standard deviation.

First we characterized prednisolone’s role in mediating NF-κB driven gene expression in human monocytic cells that were stably transfected with a TNF-α promoter driving β-lactamase, referred to as HTS-43 cells. Target engagement by the OGA inhibitor was observed in HTS-43 cells using flow cytometry as levels of O-GlcNAcylated protein. As shown in Fig 1, in HTS-43 cells, there is a clear dose dependent increase in O-GlcNAcylated protein following the inhibition of O-GlcNAcase. The EC50 of the inhibitor was 3.7 nM (Fig 1). We then examined whether OGA inhibition would result in increased cellular sensitivity to glucocorticoids. Exposure of HTS-43 cells to prednisolone resulted in a dose dependent decrease in TNF-α promoter activity, with an EC50 of 1.0 nM (95% CI: 0.7–1.7 nM) (Fig 2). Addition of thiamet-G did not enhance the potency of prednisolone (1 nM; 95% CI: 0.7–1.7 nM) (Fig 2).

We tested if increased duration of exposure to the inhibitor was required to see an effect. The results indicated that a 24 hr pre-incubation with OGA inhibitor had no sensitizing effect when compared to prednisolone alone (not shown). EC50’s for the control and 10 uM thiamet-G treatment were 2.5 nM (95% CI: 1.5–4.3 nM) and 3.4 2.6 nM (95% CI:1.7–4.0 nM), respectively. The differences in EC50 between the 30 min pre-incubation of thiamet-G vs 24 hr pre-incubation are not significant (p > 0.05). While these results did not support our hypothesis, this system only allowed us a look at one gene in a specific cell line using a recombinant reporter assay. We therefore looked to primary cells with endogenous native gene expression as the read out. This would allow us to examine across a larger gene panel in a more native system.

### Measurement of TNF-α production and gene expression profiling in human PBMCs

OGA is highly expressed in mononuclear cells [18]. Our next studies were designed to investigate the potency and efficacy of prednisolone on its ability to regulate the expression of a panel of genes in human peripheral blood mononuclear cells. The gene panel consists of 36 genes that were selected from a study conducted in human whole blood looking at genes in human whole blood treated with LPS and prednisolone (Merck unpublished data). All values were normalized to cells not treated with prednisolone to provide a top or bottom depending if the gene is transactivated or transrepressed.

**Fig 3:**
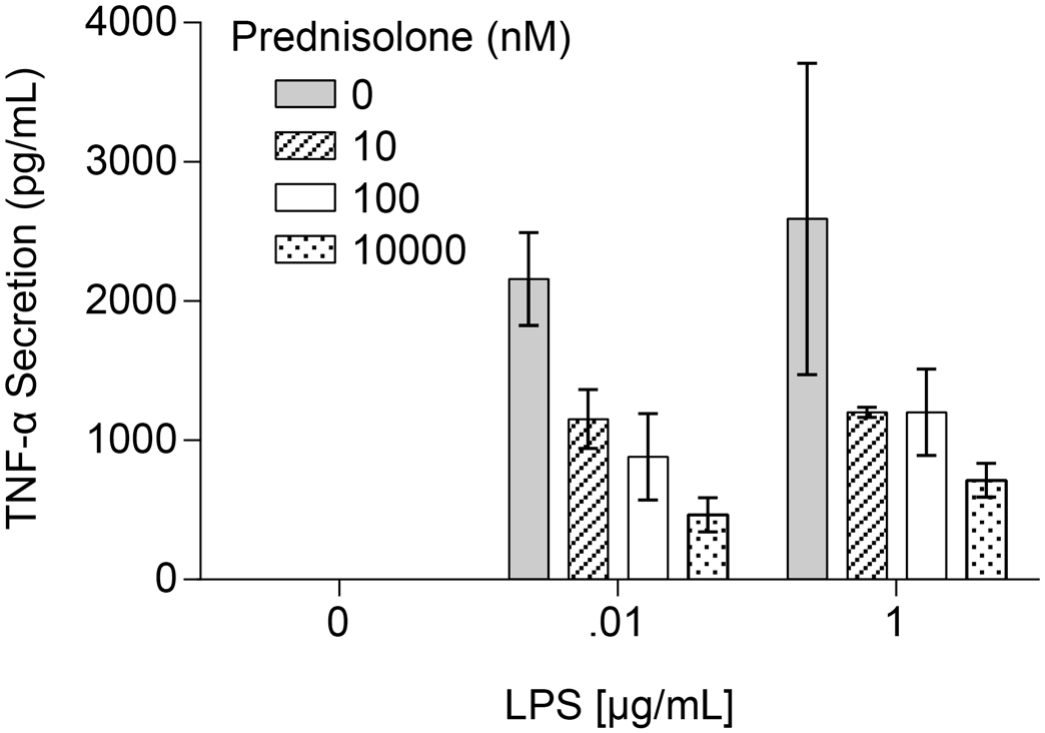
TNF-α secretion is inhibited in LPS-stimulated PBMCs treated with prednisolone. PBMCs were incubated with increasing concentrations of prednisolone (0, 10 nM, 100 nM, 10 μM) for 30 min at 37°C. Following that cells were treated with LPS (0.01 μg/mL, 1.0 μg/mL or control) for four hours at 37°C. TNF-α production was measured as described in Materials and Methods. Data points represent n = 3. Error bars represent standard deviation.

**Fig 4:**
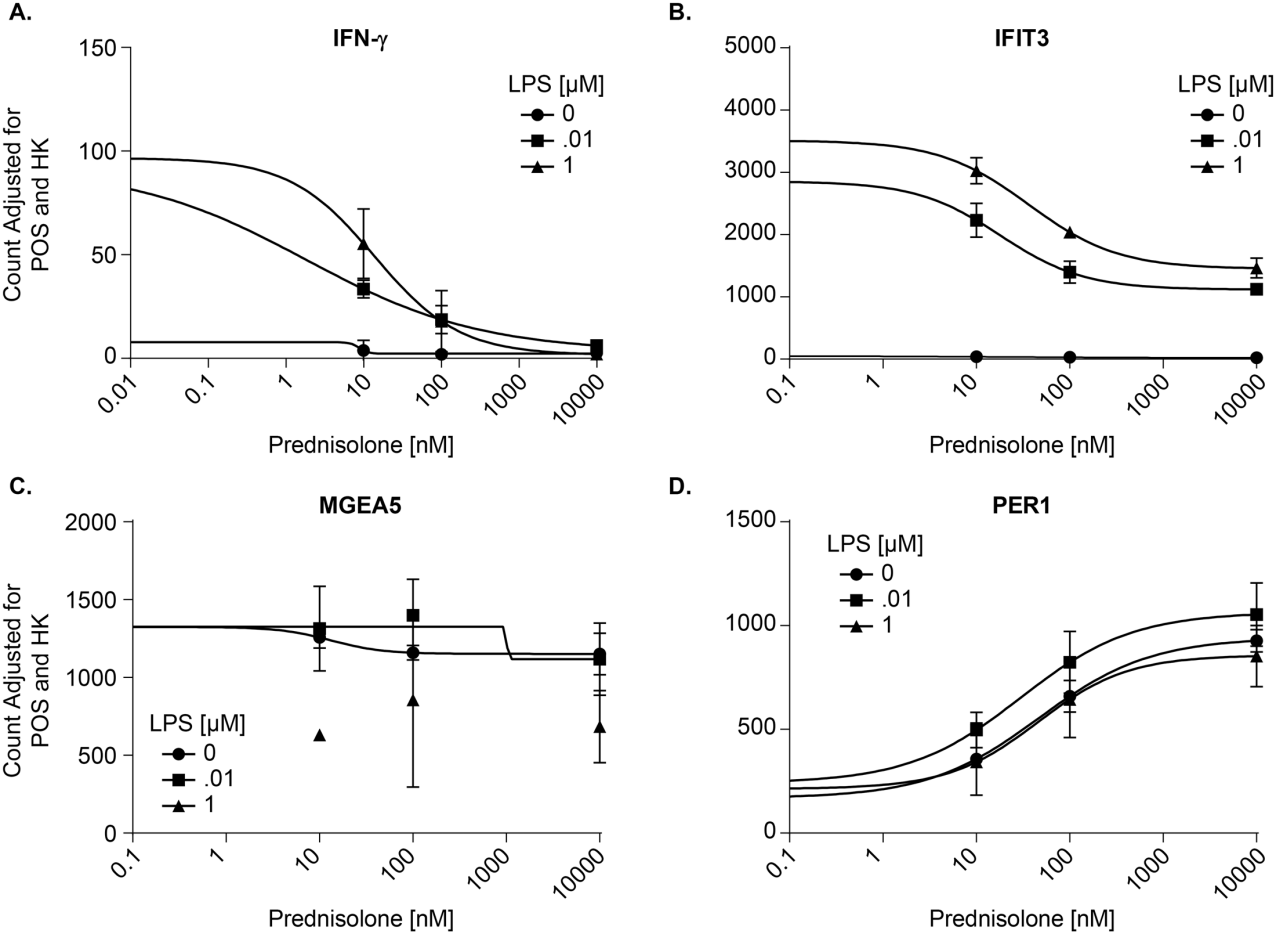
Prednisolone inhibits inflammatory genes that are upregulated by LPS in PBMCs. Human PBMCs were incubated with varying concentrations of prednisolone (10 nM, 100 nM, 10 μM or DMSO control) with LPS concentrations of 0.01 μg/mL, 1 μg/mL or vehicle. PBMCs were incubated with prednisolone for 30 min at 37°C. Cells were then stimulated with LPS for 2 hr at 37°C. Cell lysate was sent to Covance Genetis Lab (Seattle, WA) for analysis. Gene expression was analyzed in cell lysates with the NanoString nCounter system. Representative genes are used in this figure: transrepressed genes IFN-γ (a) and IFIT3 (b), transactivated gene PER1 (d) and MGEA5 as a negative control (c). Y-axis: Background correction (2) positive control correction and (3) housekeeping gene correction. The housekeeping genes were GAPDH, TUBB, GUSB, HPRT1, and PGK1. Positive control is undisclosed by nanostring. Data points represent n = 3. Error bars represent standard deviation.

Cells used for the pilot study were confirmed to be active by measuring secreted TNF-α in response to prednisolone and a challenge by LPS (Fig 3). In our pilot study, fresh PBMCs were incubated with varying concentrations of prednisolone and then treated with LPS or DMSO vehicle. Fig 4 shows representative gene data. Data for the full panel of genes is located in S1 Table. Stimulation with LPS caused a dose-dependent induction of three pro-inflammatory genes, IFN-γ (Fig 4a), IFIT3 (Fig 4b), and MGEA5 (Fig 4c), but had no effect on PER1 (Fig 4d). Prednisolone fully inhibited IFN-γ in a dose-dependent transrepression (Fig 4a). Expression of IFIT3 was dose-dependently repressed by prednisolone (Fig 4b), but the effect was incomplete even at high doses of prednisolone. This is confirmed by comparisons to baseline levels of expression in the absence of LPS. MGEA5 did not show any dose dependent regulation by prednisolone (Fig 4c). We also included genes that are transactivated by prednisolone as a control, e.g. PER1 (Fig 4d). This is based on Li’s work indicating that OGA would not impact transactivated genes [12]. The difference in responsivity is due to different promoter architectures of the genes, and different transcription factors embedded with the promoter regions with the genes. This is a well-known phenomenon in GR gene regulation. These data confirm a role for glucocorticoid regulation of gene transcription and established a model for evaluating the effects of OGA inhibition.

Target engagement of OGA by inhibitor thiamet-G was tested by measurement of accumulation of O-GlcNAc in a HTS-43 cell line, monocytes, and T cells. As expected, there is a dose dependent increase in O-GlcNAc staining in monocytes following exposure to increasing concentrations of OGA inhibitor (Fig 1). This was also observed in T cells (Fig 1). Observed EC50s were 2.9 nM and 4.6 nM for monocytes and T cells, respectively. Thus, we were confident that treating cells with 1 μM thiamet-G would pharmacologically inhibit OGA activity.

**Fig 5:**
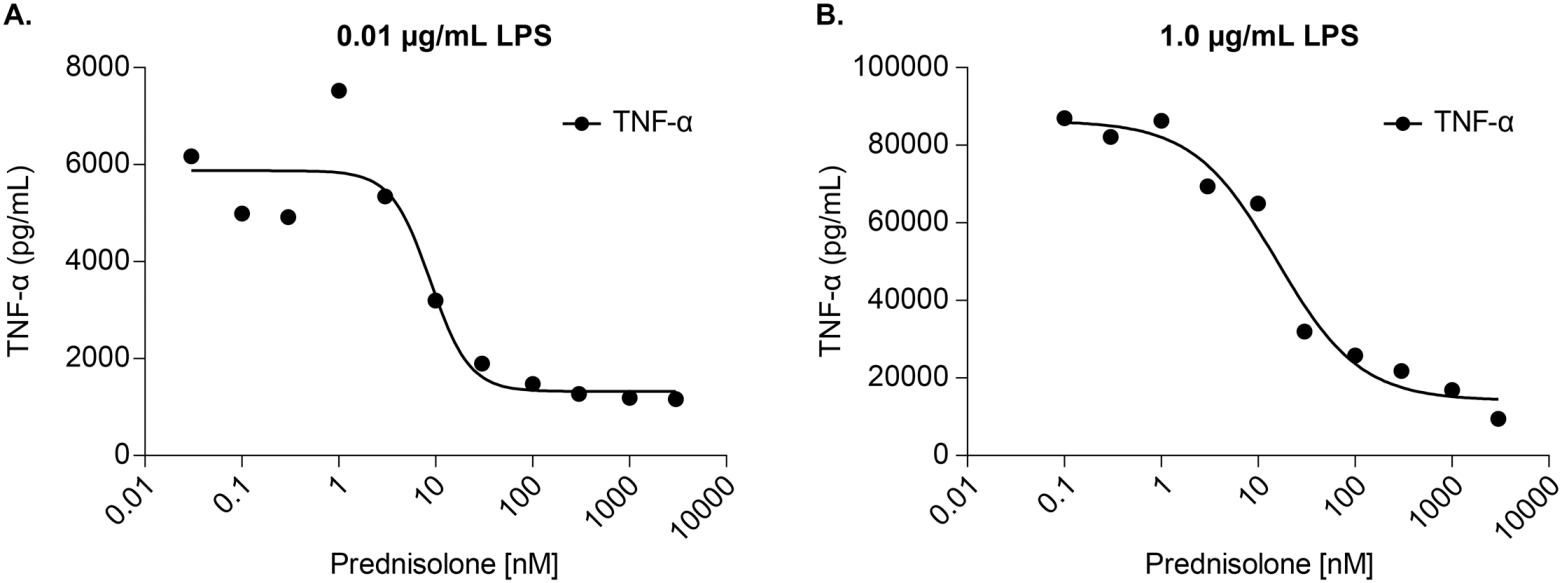
TNF-α secretion is inhibited in LPS-stimulated PBMCs treated with prednisolone. PBMCs were treated with increasing concentrations of prednisolone 30 min at 37°C. Cells were then treated with two concentrations (a) 0.01 μg/mL (b) 1 μg/mL of LPS for for four hours 37°C. Supernatant was tested for PBMC TNF-α cytokine release using a mesoscale kit. Data points represent n = 1

**Fig 6:**
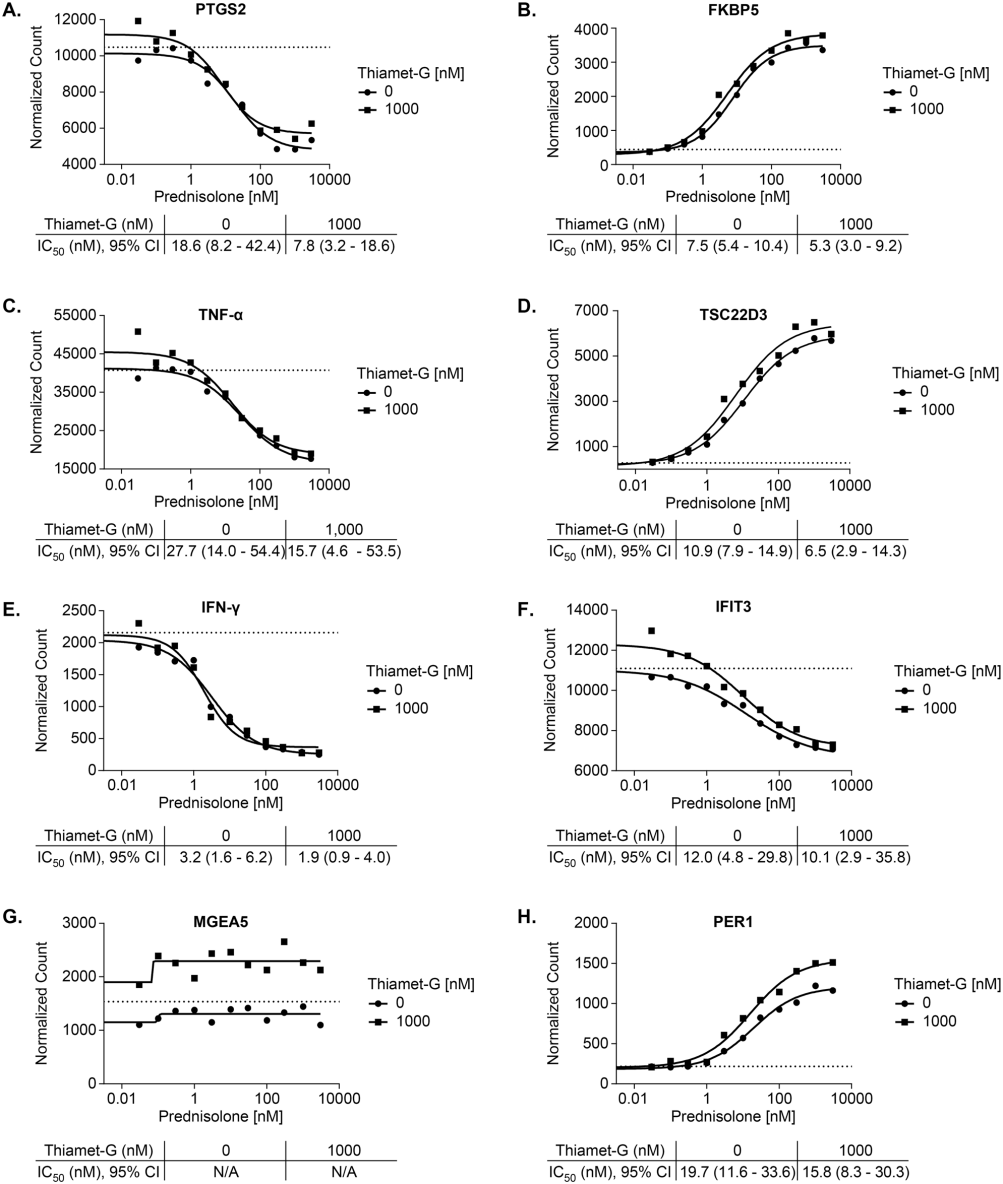
Thiamet-G does not alter potency of prednisolone in transactivated or transrepressed inflammatory genes in hPBMCs. PBMCs were incubated with a 10 point half- log titration of prednisolone starting at 3 μM and varying concentrations of OGA inhibitor for 30 min at 37°C. Cells were then stimulated with LPS for 2 hr, prior to lysis and shipment to CGL for analysis. Above is representative transrepressed genes (a) PTGS2 (c) TNF-α (e) IFN-γ (f) IFIT3 and transactivated genes (b) FKBP5 (d) TSC22D3 (h) PER1. MGEA5 has no response to prednisolone. Error of magnitude for the IC50 is represented as a 95% confidence interval. Dotted lines are the no-treatment control values. Data points represent n = 1

**Fig 7:**
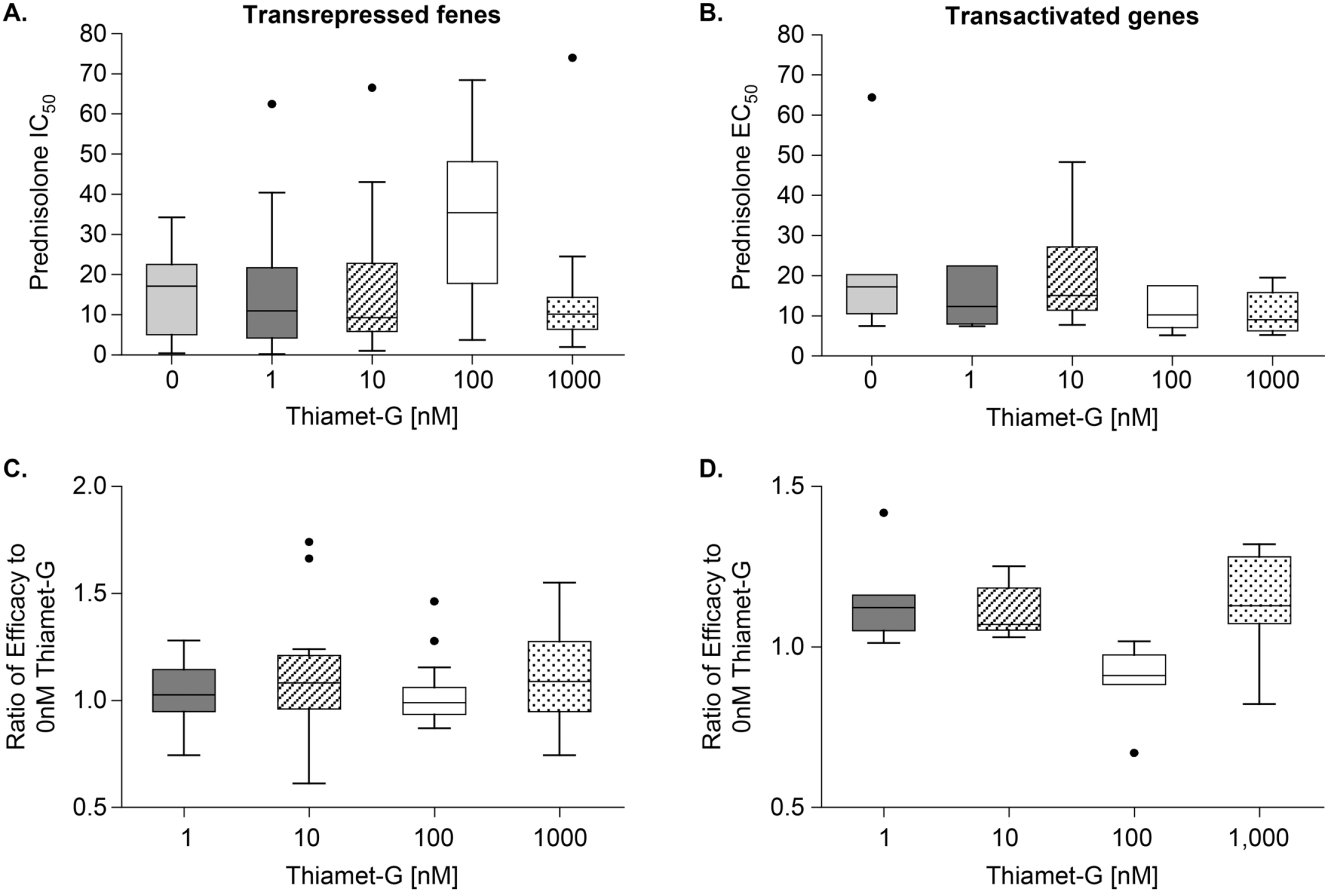
Average potency and efficacy of prednisolone are unchanged in the presence of thiamet -G in hPBMCs. Upper Panels: IC50 and EC50 values were calculated for transrepressed and transactivated genes for each OGA inhibitor dose at 1 μg/mL LPS. Average IC/EC 50 is indicated by the line in box. Thus, a lower IC50 with increasing thiamet-G dose would indicate potentiated transpression by the OGA inhibitor. Lower Panels: Efficacy was determined by taking the difference between top and bottom signals of a prednisolone dose titration curve, and the efficacy ratio represents the effect with OGA inhibitor vs the effect with OGA vehicle control. Thus, a ratio greater than 1 would indicate potentiated transpression by the OGA inhibitor. These values are plotted for all (a) transrepressed (CXCL2, IFIH1, IFIT2, IFIT3, IL1A, IL6, IL1RN, NFKBIZ, SOCS3, TRAF1, PTGS2, CD274, CD40, TNF-α, TNFSF15, IFN-γ, LTA, TRAF4) and (b) transactivated genes (FKBP5, PER1, TSC22D3, ZBTB16, GRASP, IRS2, LPL). Error bars represent standard deviation. IC50/ EC50 datapoints represent n = 1 for each gene.

The next step was to study if OGA inhibition by thiamet-G would produce a significant effect on glucocorticoid efficacy and potency in LPS-challenged PBMCs. Donor cells used for this study were confirmed to be active by measuring secreted TNF-α in response to prednisolone and challenged with LPS (Fig 5). Table 1 shows IC50 values of prednisolone in cells stimulated with two concentrations of LPS as in Fig 5, treated with prednisolone (+/- 1uM thiamet-G). Unpaired t-tests indicate differences in thiamet-G treated vs untreated are not significant in either LPS condition. Mesoscale quantification of TNF-α release over a range of prednisolone was used to determine the IC50. Representative data in Fig 6 do not suggest that the OGA inhibitor potentiated the effect of prednisolone. These genes were selected to show based on the best visualization for which to illustrate the effect of OGA inhibition on glucocorticoid potentiation. The conclusion drawn from the data however are taken as a whole. The differences in IC50/EC50 are not significant when analyzed using unpaired t-tests. Data for all transactivated and transrepressed genes in the panel are located in S2 Table. Fig 7 demonstrates that the efficacy of prednisolone response was unaffected in transrepressed genes (Fig 7a) (CXCL2, IFIH1, IFIT2, IFIT3, IL1A, IL6, IL1RN, NFKBIZ, SOCS3, TRAF1, PTGS2, CD274, CD40, TNF-α, TNFSF15, IFN-γ, LTA, TRAF4) and transactivated genes (Fig 7b) (FKBP5, PER1, TSC22D3, ZBTB16, GRASP, IRS2, LPL), as evidenced by an efficacy ratio of approximately one. Additionally, the IC50’s for prednisolone for each transrepressed gene also remained relatively unchanged in the presence of increasing thiamet-G concentrations. The EC50’s for transactivated genes also remained relatively unchanged, which was consistent with O-GlcNAc not playing a role in regulation of genes transactivated by the GR.

**Table 1:** Effect of thiamet-G on prednisolone IC50 in LPS stimulated PBMCs.

	Pred IC50 (nM)	
treatment	no thiamet-G	1 μM thiamet-G
**LPS, 1 μg/mL**	37.1 +/- 29.7	28.83 +/- 6.7
**LPS, 0.01 μg/mL**	57.3 +/- 36	51.3 +-/ 21.6

LPS, Lipopolysaccharide

### Glucocorticoid-induced apoptosis in cancer cell lines

**Fig 8:**
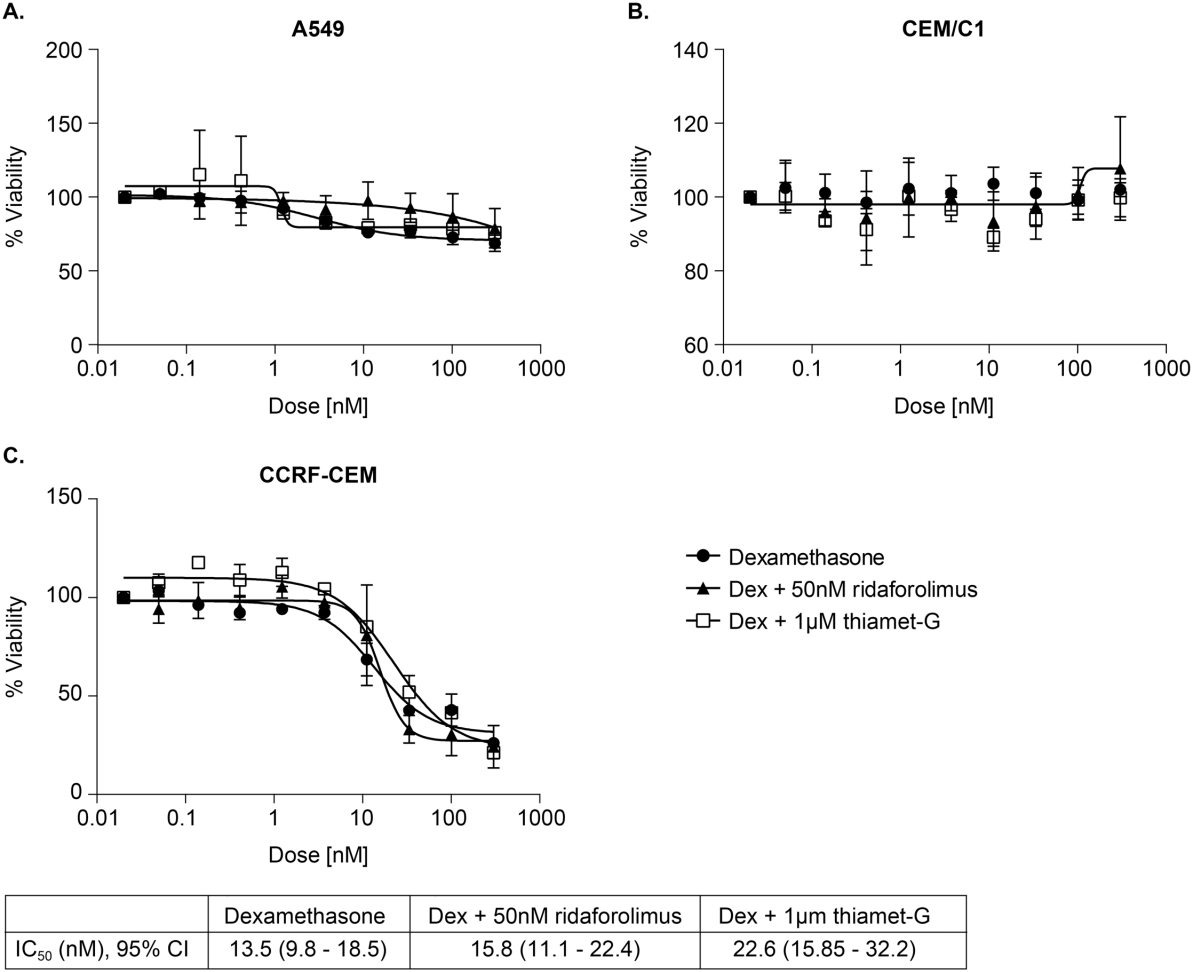
Thiamet-G does not potentiate the effect of dexamethasone-induced cell apoptosis in steroid resistant cell lines. Cancer cell lines were exposed to either only dexamethasone, or in addition to 1 μM OGA inhibitor or 50 nM ridaforolimus for 72 hr. Cell lysates were then tested using Cell Titer-Glo for a luminescent signal proportional to ATP presence. Data is represented as a percent viability from cell count from untreated cells. Error bars represent standard deviation. The calculated IC50 and 95% confidence interval are shown. Data points represent n = 3.

We wanted to determine if inhibition of OGA, by thiamet-G in A549, and CCRF-CEM and CEM-c1 would produce an effect similar to overexpression of OGT, wherein glucocorticoid–resistant cancer cells became less resistant to glucocorticoid induced cell death [12]. Ridaflorolimus was used as a sensitizing positive control. As seen in Fig 8, cell viability mlaeasurements, based on measurement of cellular ATP levels, showed that CCRF-CEM viability decreased with increasing dexamethasone treatment, but was unaffected by ridaflorolimus, and thiamet-G. A549 and CEM-c1 cells showed no change in apoptosis with the addition of dexamethasone, regardless of the presence of ridaflorolimus or OGA inhibitor. Ridaforolimus was expected to potentiate reduced cell viability of dexamethasone, but this was not the case (p = 0.99). Unpaired t-tests conclude the differences in IC50 are not significant. In addition, we monitored DNA fragmentation 18 hours after treatment with dexamethasone alone, or dexamethasone plus 1 μM thiamet-G, or dexamethasone plus 50 nm ridaforolimus, but saw no effects of any dose for any treatment (data not shown).

## Discussion

We have investigated whether inhibiting OGA using thiamet-G would modulate the sensitivity of human PBMCs or transformed cell lines to a glucocorticoid agonist. We carried out this work because of our interest in potential therapeutic targets that might offer alleviation of steroid resistance in particular in the setting of respiratory inflammatory disease. We found no evidence for such an effect, despite clear evidence for target engagement. More specifically, we showed that the potent and specific OGA inhibitor thiamet-G caused a dose-dependent increase in the levels of O-GlcNAcylated protein in cells, and then carried out functional experiments using doses of thiamet-G that produced the maximum achievable effect. Therefore we were able to exclude the possibility that the compound was not cell penetrant or not used at a high enough concentration as a basis for the lack of effect on steroid sensitivity. We examined the dose-response profile of glucocorticoids in three systems: TNF-α production in a monocytic cell line, gene expression in PBMCs, and viability of oncogenic cell lines. In every case, OGA inhibition had no impact on the dose response profile of prednisolone or dexamethasone. Together, these data do not support further investigation of OGA as a therapeutic target for improving steroid sensitivity in inflammatory disease.

The hypothesis tested in our studies was formulated based on the work of Li and co-workers which pointed to O-GlcNAcylation of proteins by OGT, probably RNA polymerase II, as part of the mechanism of repression of gene expression by the glucocorticoid receptor, and the reported reciprocity with OGA based on the review literature [12]. Our finding that inhibition of OGA had no impact might call into question the validity of the findings of Li and co-workers, but this is not our interpretation. We did not seek to reproduce the data of Li et al., and none of our findings are incompatible with the previous work. Rather, we conclude only that there is not a rate-limiting role for removal of O-GlcNAc from the potential protein that is modified by OGT that is recruited by the GR to the transcriptional complex, if that is indeed the mechanism underpinning the findings of Li’s group. It could be that OGT provides some structural rather than enzymatic role in the transcription complex such that O-GlcNAc modification is actually not part of the regulatory mechanism, and that would be consistent with our findings. At the same time, we did not measure O-GlcNAc levels on RNA polymerase II or any specific protein, just the total cellular content, and it might be that O-GlcNAcylated RNA polymerase II is somehow sequestered and protected from the action of OGA such that inhibition of OGA has no impact. In short, we have no reason to question the validity of the experimental findings of Li et al. at present. Ranuncolo’s group however supports findings from Li’s group as the former’s model suggest that in the event of excess OGT, the equilibrium pushes toward an O-GlcNAcylated state [11]. Using shRNA inhibition in cell-free and in vitro, Ranuncolo’s group found that inhibition of OGT or OGA reduced efficient pol II mediated transcription. We did not find that OGA inhibition potentiated transrepression by the GR, thus OGA may not be a general regulator of transcription, or may be less important in GR regulation of pol II.

A limitation of our work is that the cells were not induced into a glucocorticoid resistant state, and so there may not be much opportunity for a gain in glucocorticoid sensitivity [19–22]. An example of this can be seen in a study by Mercado, et al., where glucocorticoid insensitivity was induced in U937 cells that were treated with either H_2_0_2_ or cigarette smoke extract, measured by the increasing phosphorylation of Akt, as a result PI3Kδ activation. Preincubation with nortriptyline prevented the cigarette smoke extract (CSE), or H_2_0_2_-induced phosphorylation of Akt in U937. They had also measured budesonide sensitivity by evaluating its ability to inhibit TNF-α induced IL-8. When U937 cells were pre-treated CSE prior to the addition of budesonide, inhibition of IL-8 production was less effective; however, if the culture was treated with nortriptyline before CSE exposure, the efficacy of budesonide was restored [19]. However useful these models would be, they are complex, and our plan was to engage in such work only if the above studies provided supportive evidence.

Although our main focus was regulation of inflammatory genes, we also evaluated the effect of OGA inhibition on the growth of transformed cell lines as previously published [12, 23–26]. Li’s group found that, in CCRF-CEM cells, a cell line sensitive to glucocorticoid induced apoptosis, the inhibition of OGT would reduce glucocorticoid sensitivity, whereas an increase of OGT would increase cell death in A549 cells, an insensitive cell line. This suggested a role for OGT in modulating sensitivity to dexamethasone-induced apoptosis in acute lymphatic leukemias and solid tumors, the latter of which are generally resistant to glucocorticoid-induced cell death [12, 27]. Inhibition of mTOR has shown to increase the pro-apoptotic activity of glucocorticoids and Lamb’s group demonstrated that treatment of CEM-c1 cells with sirolimus, an inhibitor of the mTOR pathway, resulted in increased cell sensitivity to dexamethasone [25]. This has also been seen in work done by Gu and coworkers in T-ALL cells using rapamycin, another mTOR inhibitor [23]. Resistance to apoptosis in CEM-c1 cells were also shown to be reversed with the combination of dexamethasone and forskolin, an activator of the protein kinase A [28]. We used this literature precedent to bridge our work with the findings gathered by Li and others by working within their established methods for OGT’s role in cancer cell sensitivity to glucocorticoid-induced apoptosis. We hypothesized that OGA inhibition would increase cell sensitivity in resistant cell lines, similar to OGT overexpression. CEM-c1 and the cell sensitizer ridaforolimus (an mTOR inhibitor) were used as a positive control. Our data do not support a role for OGA inhibition in modulating glucocorticoid-induced cell death. One possibility for the discrepancy is the difference in the methodology used. Li’s group used the overexpression of OGT, a non-native system, whereas we have used pharmacological inhibition in an unmodified cellular system. However it is worth noting that previous studies have shown that in many oncogenes and tumor suppressors, OGT and O-GlcNAc levels are elevated and that O-GlcNAcylation plays a role in breast cancer metastasis [29–31]. It can aid in anchorage-independent growth of colon and lung cancers, including A549, where the use of thiamet-G alone against A549 actually increased its colony formation ability [26]. These findings from other groups could help explain the lack of sensitivity to dexamethasone in the cell lines we have tested, versus what Li’s group had found. If it is true that OGT is more active and the level of O-GlcNAcylated oncogenes are elevated in transformed cell lines, inhibition of OGA might promote cell survival. However, assuming OGT was not elevated, and the accumulation of O-GlcNAc by OGA inhibition was significant, it would cause elevation in O-GlcNAcylated oncogenes, again promoting cell survival. In the case where the CCRF-CEM cells showed decreased viability when treated with dexamethasone, it is unclear whether the cells in our study became apoptotic. It is possible that the cells that were exposed to higher concentrations of dexamethasone had growth arrested, while cells exposed to lower concentrations of dexamethasone continued to proliferate. A complimentary experiment using ELISA cell death detection showed no response to dexamethasone in any cell line. Neither was there any effect by the OGA inhibitor observed in either apoptosis study. Further studies might include measuring relative levels of OGT and O-GlcNAc between PBMCs and cancer cell lines we used, based on information from previous work, along with testing of other sensitizers that we have not used.

While we did not to find evidence of a therapeutic role for OGA inhibition in regard to steroid sensitivity, inhibition of OGA by thiamet-G still offers a number of potential therapeutic uses. Abnormal hyperphosphorylation of microtubule-associated protein tau, which can also be O-GlcNAcylated, plays a crucial role in neurodegeneration in Alzheimer's disease and it’s been shown that treatment with thiamet-G led to a decrease in tau phosphorylation and slowing of neuro degeneration [15–17]. Inhibition of OGA by thiamet-G has also been shown to prevent inflammation-induced vascular dysfunction [32].

In conclusion, we tested but did not substantiate the hypothesis that inhibition of OGA would increase the sensitivity or efficacy of glucocorticoids with respect to repression of inflammatory gene expression.

## Supporting Information

S1 Table Full gene panel data to supplement Fig 4. (XLSX)

S2 Table Full gene data to supplement Fig 6, featuring transactivated and transrepressed genes. (XLSX)
